# Efficacy and safety of pembrolizumab as first-line treatment for advanced non-small cell lung cancer complicated with chronic obstructive pulmonary disease: protocol for a prospective, single-arm, single-center, phase II clinical trial

**DOI:** 10.3389/fonc.2024.1179232

**Published:** 2024-03-07

**Authors:** Weigang Dong, Yan Yin, Bin Liu, Yan Jiang, Lei Wang, Dongsheng Shi, Jianwen Qin

**Affiliations:** Department of Respiratory and Critica Care Medicine, Tianjin Chest Hospital, Affiliated Chest Hospital of Tianjin University, Tianjin, China

**Keywords:** chronic obstructive pulmonary disease, pembrolizumab, efficacy, safety, non-small cell lung cancer (NSCLC)

## Abstract

**Background:**

The first-line standard treatment option for patients with NSCLC complicated with Chronic obstructive pulmonary disease (COPD) is still unclear and relies on the treatment option of NSCLC alone. To date, a limited number of retrospective studies have explored the efficacy and safety of immunotherapy in patients with NSCLC complicated with COPD. We therefore designed this study to further explore the efficacy and safety of first-line immunotherapy in patients with NSCLC complicated with COPD.

**Methods:**

This study was designed as a single-armed, single-center, prospective, phase II clinical study. It will include 30 advanced (stage IV) NSCLC combined with COPD primary treatment subjects. Each subject’s diagnosis will be confirmed by clinical, radiographic, pathologic, and pulmonary function evaluation. A fixed dose of 200 mg pembrolizumab will be administered by intravenous infusion on day1 every 3 weeks (Q3W). The management of stable and acute exacerbations of COPD include home oxygen therapy, and the use of conventional medications are also administered. Imaging evaluation will be performed every 6 weeks for 6 months from the first pembrolizumab dose and approximately every 12 weeks thereafter until disease progression or early withdrawal. COPD status will be evaluated every 3 months by pulmonary function, GOLD grading, mMRC score, CAT score, ABCD grouping, and AECOPD severity. The primary outcome is Progression-free survival. The secondary outcome measures include objective response rate, overall survival, rate of acute exacerbations of COPD (times/year), lung function, mMRC score, CAT score, impact of treatment on patient’s health-related quality of life, antibiotic use (including duration and classes), and adverse events associated with immune checkpoint inhibitors. Exploratory endpoint is to explore the association between COPD grade and the degree of immune cell (CD4+ T lymphocytes and CD8+ T lymphocytes) infiltration, as well as the association between COPD grade and the efficacy of immune checkpoint inhibitors.

**Clinical trial registration:**

ClinicalTrials.gov, identifier NCT05578222.

## Introduction

Chronic obstructive pulmonary disease (COPD) is the third leading cause of death, and lung cancer is the second most common type of cancer worldwide. Both have high global incidence and mortality rates, which significantly increase socioeconomic burden (1–3). Smoke exposure, family history (genetic predisposition), and chronic inflammation are common risk factors for COPD and lung cancer (4), with smoking being the leading risk factor for lung cancer and COPD. COPD is associated with an increased lung cancer development, which is often underestimated in clinical practice. Studies have reported that 40-70% of lung cancer cases are complicated with COPD (5, 6).

COPD affects the incidence of lung cancer and may also influence the efficacy of cancer therapies (7, 8). An abnormal inflammatory response plays a role in the occurrence and development of both diseases. The tumor microenvironment of lung cancer is conducive to angiogenesis and immunosuppression, which eventually leads to the immune escape of tumor cells and tumor formation (9, 10). COPD-like chronic inflammation is associated with immune escape mediated by the programmed death-1 (PD-1)/programmed cell death 1 ligand 1 (PD-L1) pathway and has been shown to be involved in the occurrence and development of COPD-associated lung cancer (10). In addition, in a COPD mouse model complicated with lung cancer, chronic inflammation negatively affected the efficacy of immune checkpoint inhibitors (10, 11).

Pembrolizumab is a humanized monoclonal antibody against PD-1 used in cancer therapy. To date, five randomized clinical trials have demonstrated the substantial efficacy of pembrolizumab in non-small cell lung cancer (NSCLC). The Phase III KEYNOTE-024 trial showed that first-line pembrolizumab monotherapy improved progression-free survival (PFS, 10.3 vs. 6.0 months, hazard rate [HR] = 0.50) and overall survival (OS; 30.0 vs. 14.2 months, HR = 0.63) when compared with chemotherapy. In addition, pembrolizumab treatment resulted in fewer adverse events (AEs) among patients with metastatic NSCLC without EGFR/ALK alterations and a PD-L1 tumor proportion score of 50% or greater (12). KEYNOTE-042 expanded the inclusion criterion to PD-L1 tumor cell proportion score (TPS) ≥ 1%, and the results suggested that pembrolizumab significantly reduced the risk of death by 19% compared with chemotherapy. However, subgroup analysis suggested that the main beneficiary group was patients with PD-L1 TPS ≥ 50% (13). The results of thesubgroup analysis showed that compared with chemotherapy, pembrolizumab significantly prolonged the OS of patients with PD-L1 TPS ≥ 50% (median 20.0 vs. 14.0 months, HR = 0.62) and those with PD-L1 TPS 1–49% (median 19.9 vs. 10.7 months, HR = 0.69) (14). In 2019, the National Medical Products Administration approved pembrolizumab as the first-line treatment for patients with PD-L1 TPS ≥ 1%. According to the Chinese Society of Clinical Oncology guidelines, pembrolizumab is recommended for the first-line treatment of advanced driver negative NSCLC (Grade I recommendation), with PD-L1 TPS ≥ 50% as class 1A evidence and PD-L1 TPS ≥ 1% as class 2A evidence.

However, the KEYNOTE series of NSCLC-related studies did not consider COPD comorbidities. COPD patients often have a history of smoking. El-Osta et al. (15) found that smoking has been linked to increasing the immunogenicity of tumor cells and may improve the efficacy of immunotherapy. In addition, Malek AE et al. (16) have shown that COPD and smoking are independently associated with an increased risk of infection in patients treated with immune checkpoint inhibitors. Therefore, the efficacy and safety of pembrolizumab as a first-line treatment of patients with advanced NSCLC complicated with COPD are unclear. Retrospective studies have shown that the efficacy of PD-1/PD-Ll immune checkpoint inhibitors in patients with COPD combined with lung cancer is better than that in patients with lung cancer alone (17–19). However, these studies have some limitations. First, known clinical data were obtained retrospectively, and there are many confounding factors that may affect the results, such as age, pathological type of NSCLC, COPD grade, smoking status, and hormone use. Second, the influence of COPD on immune cell infiltration in tumor tissue is unclear. A study by Biton (17) showed that tumor-specific CD8+ T cell depletion positively correlated with COPD severity. However, Mark et al. (18, 20, 21) found that the infiltration level of tumor-specific CD4+ and CD8+ T cells in patients with COPD complicated with lung cancer was higher than that of patients without COPD. In addition, findings regarding the correlation between COPD and PD-L1 expression in NSCLC tissues are conflicting. Third, previous studies have suggested that PD-1/PD-Ll immune checkpoint inhibitors could aggravate lung tissue damage and COPD symptoms (19, 22). However, these studies lacked parameters, such as the GOLD scale, mMRC dyspnea questionnaire, CAT score, and ABCD grouping, which limited COPD assessment. Therefore, in this clinical trial, we aim to evaluate the efficacy and safety of pembrolizumab as a first-line treatment for advanced NSCLC with COPD.

## Methods

### Study design

**Figure 1:**
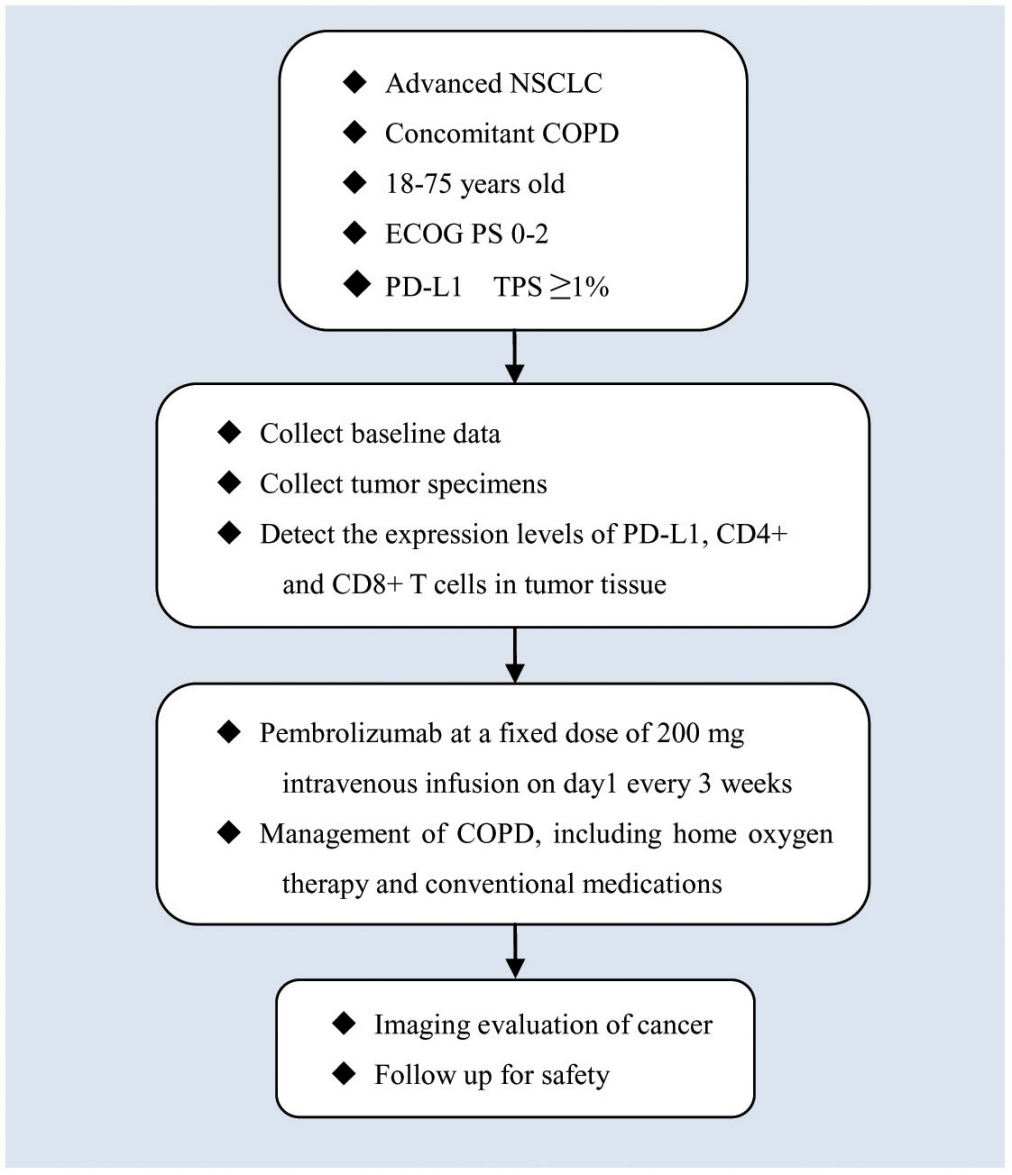
A schematic diagram of subjects’ enrolment, interventions, assessments, and visits.

This study was designed as a single-arm, single-center, prospective, phase II clinical study. It will include 30 advanced (stage IV) NSCLC combined with COPD primary treatment subjects. The diagnosis of each subject will be confirmed by clinical, radiographic, pathologic, and pulmonary function evaluation. Patient recruitment was initiated in November 2022, and we plan to complete the study in December 2024. This trial has been approved by the Institutional Review Board of Tian Chest Hospital (Approval date: August 25, 2022 and approval number: 2022KY-012-01) and has been registered in the clinicaltrials.gov database (NCT05578222). A schematic diagram of the trial is presented in **Figure 1**.

### Eligibility criteria

Adult patients with treatment-naïve, confirmed advanced (stage IV) NSCLC combined with COPD, PD-L1 TPS ≥ 1%, and an Eastern Cooperative Oncology Group performance status of 0 to 2 will be enrolled. As mentioned previously, NSCLC stage and diagnosis will be confirmed by clinical, radiographic, pathologic, and pulmonary function evaluation. Inclusion and exclusion criteria are shown in **Table 1**.

**Table 1:** Inclusion and exclusion criteria.

Key inclusion criteria
Ø Signed written informed consent before enrollment; Ø Men and women aged 18-75; Ø The patient must be able to provide fresh or archived tumor tissue and its pathology report; Ø No previous antitumor therapy, life expectancy is not less than 12 weeks; Ø Newly diagnosed NSCLC subjects confirmed by pathology and imaging, Stage: cT1-4N1-3M1 (stage IV), AJCC eighth edition clinical staging of lung cancer; Ø Asymptomatic patients with NSCLC brain metastases; Ø PD-L1 tumor fraction (TPS) ≥1%; Ø Epidermal growth factor receptor (EGFR) gene mutation negative and anaplastic lymphoma kinase (ALK) negative; Ø Measurable lesions that meet RECIST v1.1 Criteria; Ø Patients with COPD who met the relevant diagnostic criteria for COPD in the Guidelines for the Diagnosis and Treatment of Chronic Obstructive Pulmonary Disease (Revised 2021) formulated by Chinese Society of Respiratory Medicine, and whose condition was stable for ≥2 weeks; Ø ECOG: 0 ~ 2; Ø The functions of vital organs meet the following requirements (excluding the use of any blood components and cell growth factors within 14 days): Normal bone marrow reserve, neutrophil (ANC) ≥1,500/mm³, platelet count (PLT) ≥100,000/mm³, hemoglobin (Hb) ≥5.6mmol/L (9g/dL); Creatinine (Cr) ≤1.5 mg/d and/or Clearance of creatinine (CCr) ≥60 ml/min; Normal liver function or Total Bilirubin (TBIL) ≤1.5ULN, Aspartate Transaminase (AST) and Alanine Transaminase (ALT) ≤1.5 ULN; Ø Non-surgical sterilization or use of a medically approved contraceptive method (e.g. intrauterine device, birth control pill, or condom) during the study treatment period and for 3 months after the end of the study treatment period. Patients with non-surgical sterilization or women of childbearing age had to have a negative serum or urine HCG test within 7 days before study enrollment; Must be non-lactation period; Male patients who are not surgically sterilized or of childbearing age are required to consent to use a medically approved contraceptive method with their spouse during the study treatment period and for 3 months after the study treatment period; Ø Subjects volunteered to join this study, with good compliance, safety and survival follow-up.
Key exclusion Criteria
Ø Previous radiotherapy, chemotherapy, long-term or high-dose hormone therapy, surgery, or molecular targeted therapy; Ø The subject has previous or concurrent other malignant tumors; Ø History of asthma or other respiratory diseases (bronchiectasis, tuberculosis, interstitial pneumonia, occupational lung disease, sarcoidosis, etc.); Patients with objective evidence of radiation pneumonitis, drug-associated pneumonitis, or severe impairment of pulmonary function; Ø History of lobectomy; Ø A history of clinically significant circulatory failure; Ø Significant contraindications for spirometry (history of myocardial infarction, cerebral artery or aortic aneurysm, history of eye surgery, hemoptysis); Ø Patients with rheumatic diseases; Patients with heart, cerebrovascular, liver, kidney, hematopoietic system and other serious primary diseases; Ø Patients with a history of acute gastrointestinal bleeding within 3 months; Ø Previous immune checkpoint inhibitor therapy; The subject is known to have a previous allergy to macromolecular protein preparations, or to any PD-1 mab component; Ø The subject has any active autoimmune disease or a history of autoimmune disease (e.g., not limited to autoimmune hepatitis, interstitial pneumonia, uveitis, enteritis, hepatitis, hypophysitis, vasculitis, nephritis, hyperthyroidism, hypothyroidism; Subjects with vitiligo or complete remission of asthma in childhood without any intervention as adults were included; Subjects with asthma requiring medical intervention with bronchodilators were excluded); Ø The subject has active infection or unexplained fever >38.5 ℃ during the screening period or before the first administration; Ø Subjects with innate or acquired immune deficiency, such as HIV infection, or active hepatitis (transaminases did not meet the inclusion criteria, HBV DNA≥10^4^/ml, HCV RNA≥10³/ml); Chronic hepatitis B virus carriers, HBV DNA≥2000 IU/ml (≥10^4^copies/ml), must receive antiviral therapy during the trial to be enrolled; Ø The subject is participating in another clinical study or it has been less than one month since the end of the previous clinical study; Subjects may receive other systemic antitumor therapy during the study; Ø Receive live vaccine less than 4 weeks before or during the study period possibly; Ø The subject cannot or does not agree to pay for the examination and treatment expenses at his own expense; Ø Pregnant or lactating women; Ø Other conditions that should be excluded from the study in the opinion of the investigator

### Patient withdrawal

Patients will be withdrawn from this study for the following reasons: (1) Subject or legal representative requests early withdrawal; (2) Unexpected rapid progression or radiographically confirmed disease progression occurs; (3) COPD-related exacerbations progress to acute exacerbation of chronic obstructive pulmonary disease (AECOPD) grade II-III; (4) Occurrence of any of the following during treatment: grade 3 hematologic toxicity, grade 3 neutrophil count decrease with fever (>38.5°C), grade 3 thrombocytopenia with bleeding tendency, esophageal perforation, severe pulmonary/mediastinal infection, hemorrhage, myocardial infarction, heart failure, severe arrhythmia, grade 2 or higher grade immune pneumonia, grade 3 or higher grade immune hepatitis, infusion reactions, or other immune-related toxicity; (5) 2-week treatment delay (for any reason); (6) Pregnancy; (7) The investigator considers it necessary to withdraw a patient from the study; (8) Subject cannot be followed up. All patients who withdraw will be followed up according to the study protocol, and the follow-up results will be recorded unless the patient withdraws informed consent and refuses to follow-up.

### Study treatments

The management of stable and acute exacerbations of COPD include home oxygen therapy and the use of conventional medications such as bronchodilators, inhaled corticosteroids, and expectorants.

A fixed dose of 200 mg pembrolizumab will be administered via intravenous infusion on day 1 every 3 weeks (Q3W). The efficacy will be evaluated according to the Response Evaluation Criteria in Solid Tumours (RECIST) v1.1 evaluation criteria. If a progressive disease (PD) result is obtained, medication use will be continued in patients with clinically stable symptoms and in patients that could potentially benefit from continued treatment. Efficacy will then be re-evaluated after 4 weeks using the immune-related response criteria (irRC) criteria. If the reassessment result is no progression, treatment will be continued. Treatment will also be continued (with consent) if the result is progression, the subject has no other treatment options, or the investigator believes that the patient would benefit from continued treatment. Patients will be withdrawn from the study upon their or the investigator’s request or owing to PD or intolerable toxicity.

Drugs that interfere with hepatic P450 enzymes and cause QT prolongation in the heart, traditional Chinese medicines, and immunological agents with anticancer properties will be contraindicated or used with caution during the study period. At the end of the treatment period, the investigator will recommend follow-up treatment.

### Outcome measurements

The primary outcome is PFS, defined as the time from randomization initiation to the progression of tumor or death for any reason (refer to RECIST v1.1).

The secondary outcomes include objective response rate (ORR), overall survival (OS), rate of acute COPD exacerbations (times/year), lung function, mMRC score, CAT score, impact of treatment on patient’s health-related quality of life (HRQoL), antibiotic use (including duration and classes), and AEs associated with immune checkpoint inhibitors. ORR is defined as the proportion of patients with complete response (CR) and partial response (PR) and is used to estimate the treatment efficacy according to RECIST v1.1. OS was measured as the period from the diagnosis of lung cancer to death. Acute exacerbation was defined if respiratory tract symptoms such as cough, sputum production, and dyspnea were significantly worse than usual, and the treatment program had to be adjusted, such as by using oral or intravenous glucocorticoid treatment combined with or without antibiotics, while excluding pneumothorax, heart failure, pneumonia, and other diseases. Acute exacerbations of COPD are classified as Grades I-III according to clinical symptoms.

Exploratory endpoint is to explore the association between COPD grade and the degree of immune cell (CD4+ T lymphocytes and CD8+ T lymphocytes) infiltration, as well as the association between COPD grade and the efficacy of immune checkpoint inhibitors.

### Study schedule and assessments

The study flow and visit schedule are shown in **Table 2**. Baseline data, such as ECOG score, QLQ-C30 score, height, weight, physical examination, vital signs, laboratory examination, electrocardiogram, lung function, mMRC score, CAT score, ABCD grouping, and AECOPD severity, will be recorded during the screening period (less than 2 weeks prior to initial administration of the study drug). Previous and recent tumor samples will be collected. The expression of PD-L1 in tumor tissue samples will be determined via immunohistochemical staining, and the expression of CD4+ and CD8+ T lymphocytes will be determined via immunofluorescence staining.

**Table 2:** Study flow chart and visit schedule.

FLOW CHART	Screening	Treatment visit	End of treatment visit	Safety follow-up visit
Timepoint	-d14–d0	Before dosing every cycle	Within 7 days after termination of treatment	Within 30 days after the last dose
Informed consent	X			
Inclusion and Exclusion criteria	X			
Demographic characteristics	X			
Medical history and treatment history	X			
Physical examination	X	X	X	X
ECOG score	X	X	X	X
QLQ-C30 score	X	X	X	X
Vital sign	X	X	X	X
Tumor tissue sample	X			
Virus detection:HbsAg,HbsAb,HbcAb,HCVAb,HIVAb	X			
Complete blood count, Urine Routine Examination	X	X	X	X
Alanine aminotransferase, Aspartate aminotransferase, Direct bilirubin, Total bilirubin, Glucose, Potassium,Sodium,Calcium, Phosphorus, blood urea nitrogen,Creatinine,Creatinine clearance, Total cholesterol	X	X	X	X
Activeated partial thromboplastin time, Prothrombin time, Thrombin time,International Normalized Ratio	X	X	X	X
Pregnacy test	X		X	
Free triiodothyronine, Free thyroxine, Thyroid-stimulating hormone	X	X	X	X
Cortisol, Adrenocorticotropic hormone	X	X	X	X
12-lead electrocardiogram	X	X	X	X
Echocardiography	X			
Lung function	X	Once every 3 months	X	
Evaluation of COPD symptoms	X	Once every 3 months	X	
Risk assessment of COPD exacerbations	X	Once every 3 months	X	
Evaluation of severity of acute exacerbation of COPD	X	Once every 3 months	X	
Drug administration		X		
Chest and abdominal CT (or abdominal and adrenal ultrasound), Cranial enhanced MRI,Neck ultrasound, Bone ECT, PET-CT	X	X		
Adverse events and drug combinations	X	X		
Survival follow-up		X		

Baseline tumor imaging evaluation will be completed within 2 weeks of initial treatment. Imaging evaluation will be performed every 6 weeks for 6 months from the first pembrolizumab dose and approximately every 12 weeks thereafter until disease progression or early withdrawal. The anti-tumor efficacy will be evaluated according to RECISTv1.1 and irRC (23).

Pembrolizumab-related AEs will be determined after each treatment cycle. The severity of AEs will be determined according to NCI-CTC AE 5.0. Investigators will be responsible for the appropriate measurement of AEs and will determine the relationships between AEs and the study drugs. AEs will be followed up until they were grades 0 to 1 or stabilized or returned to baseline levels. COPD status will be evaluated every 3 months according to pulmonary function, GOLD grading, mMRC score, CAT score, ABCD grouping, and AECOPD severity (24).

HRQoL will be assessed by completing the European Organisation for Research and Treatment of Cancer core quality of life questionnaire (QLQ-C30) at baseline, evaluation visits, end-of-treatment visits, and safety follow-up visits (within 30 d after the last dose).

### Data management

A paper case report form (CRF) will be developed before commencing the study. Patient information will be recorded using the CRF. The data manager will verify the CRF data, which will then be entered into a data management system. Doubtful data will be provided to the researchers for verification. Patient-identifying information, such as name, telephone number, and home address, will not be entered into the data management system. The verified data will be sent to statisticians for statistical analysis.

Some useful measures are often used to minimize the occurrence of lost follow-up. Firstly, a clinical nurse will be arranged to maintain long-term contact with the patien, and save information on commonly used contact methods of the patient such as telephone number, WeChat, email address, among others. In addition, the nurse will be in contact with the patient every other day to monitor the patient for immune-related adverse reactions. When the patient reports an adverse reaction, the nurse will promptly report it to the doctor, who will then contact the patient and give reasonable treatment. At the same time, it is also very important to save the contact information of at least one family member of the patient, so that the patient can be contacted in a timely manner.

### Statistics

#### Determination of sample size

Based on the data from the KEYNOTE-042 study, the median PFS of the target subjects receiving the trial treatment in this study is expected to be 6 months. The enrollment time is 1.5 years, and the follow-up time is 1 year. The type I error rate is 5%, and the power is 80%. Considering a 10% abscission rate, the current study will include 30 patients with advanced NSCLC with COPD.

#### Planned analyses

The mean, standard deviation, median, quartile, and maximum and minimum values will be calculated for measurement data. Frequency and percentage will be calculated for counting data. Paired-samples t-test and the Wilcoxon rank sum test will be performed to compare before- and after-treatment measurements for normal and non-normal distributions, respectively. The Kaplan-Meier method will be used to estimate the median progression-free time and its 95% confidence interval. Full analysis set (FAS) and per protocol set (PPS) will be used to analyze the efficacy indexes, and safety set (SS) will be used to analyze the safety data. Statistical significance will be set at p-value < 0.05.

#### Trial status

This trial is open for recruitment. The first patient was enrolled in February 2023, after all legal approvals required in China were received.

## Discussion

Currently, immune checkpoint inhibitors are the standard first-line treatment for patients with stage IV driver-negative NSCLC. However, evidence-based medical use of first-line immunotherapy for NSCLC does not consider COPD, which may affect the efficacy and safety of the treatment. Currently, the first-line standard treatment option for patients with NSCLC complicated with COPD remains unclear and relies on the treatment of NSCLC alone. To date, a limited number of retrospective studies have explored the efficacy and safety of immunotherapy in patients with NSCLC complicated with COPD. Therefore, this prospective, single-arm phase II clinical study was designed to explore the efficacy and safety of first-line immunotherapy in patients with NSCLC complicated with COPD. We expect that the results of this phase II study will provide preliminary evidence for future randomized controlled clinical trials on the use of first-line immunotherapy for NSCLC complicated with COPD.

## Ethics statement

This trial has been approved by the Institutional Review Board of Tian Chest Hospital (Approval date: August 25,2022 and approval number: 2022KY-012-01). All subjects provided written informed consent.

## Author contributions

Concept Proposal: WD and JQ. Survey and Data Summary: YJ and LW. Data Collection, analysis and statistics: YY and BL. Research Regulatory: YY. Writing - Draft: WD. Writing-Proofreading and Editing: WD and DS. All authors contributed to the article and approved the submitted version.
